# Antibiotics in dental practice: Global implications, clinical realities and the path towards stewardship

**DOI:** 10.6026/973206300221256

**Published:** 2026-02-28

**Authors:** Anuja Nimbalkar, Alaa Eltayeb, Kanika Malhotra, Arjun Sood, Harini Ramaswamy, Sowmya Upadyayula

**Affiliations:** 1 Department of Dentistry, Maharashtra University of Health Sciences, Nashik, Maharashtra, India; 2 Department of Dentistry, University of Medical Sciences and Technology, Khartoum, Sudan; 3 Department of Dentistry, Maharishi Markandeshwar University, Mullana, Ambala, Haryana, India; 4 Department of Dentistry, Pandit Bhagwat Dayal Sharma University, Rohtak, Haryana, India; 5 Department of Dentistry, SRM Kattankulathur Dental College and Hospital, Chennai, Tamil Nadu, India; 6 Department of Dentistry, Government Dental College, Vijayawada, Andhra Pradesh, India

**Keywords:** Antibiotic stewardship, dental guidelines, prophylaxis, antimicrobial resistance, odontogenic infections

## Abstract

Antibiotics play a crucial role in managing odontogenic infections, but inappropriate prescribing within dental practice still contributes to growing antimicrobial resistance. This review describes current U.S. recommendations from the Centers for Disease Control and Prevention (CDC), the American Dental Association (ADA), the American Heart Association (AHA) and the American Academy of Pediatric Dentistry (AAPD). Research supports using narrow-spectrum antibiotics for short treatment periods, while prophylactic use is now mainly reserved for patients with certain cardiac or immunocompromising conditions. Ongoing over prescription is often linked to uncertainty in diagnosis, patient pressure and limited stewardship systems. Thus, the need for more practical strategies like expanding education, clinical decision tools and interprofessional collaboration to strengthen antibiotic stewardship.

## Background:

Antibiotics are fundamental for managing odontogenic infections and preventing any systemic complications, yet their misuse in dentistry has caused some significant public health concerns [1]. Several evidence-based guidelines have been issued by the American Dental Association, American Academy of Pediatric Dentistry and Centers for Disease Control and Prevention, which emphasize conservative local treatment and reserve antibiotics for cases that show systemic spread or diffuse swelling [2]. Rough estimation suggests that dentists account for 10% of outpatient antibiotic prescriptions within the United States, with amoxicillin, amoxicillin-clavulanate, penicillin VK and clindamycin being most frequently used [3]. Generally, narrow-spectrum penicillin remains a first-line choice, thereby reserving clindamycin for β-lactam-allergic patients to minimize the risk of *Clostridioides difficile* infection [4]. The AHA and ADA recommendations restrict prophylactic antibiotics to patients with clearly defined high-risk cardiac conditions, emphasizing a selective, patient-specific approach rather than routine use [5]. Despite these recommendations, the inappropriate prescribing of antibiotics remains an issue, highlighting the continued need for stronger antibiotic stewardship efforts [6]. Stewardship initiatives adapted from the CDC's "Core Elements of Outpatient Antibiotic Stewardship" have been integrated into dental practice, promoting education, monitoring and interdisciplinary collaboration with the objective of mitigating these effects [7]. Therefore, it is of interest to describe the current U.S. guidelines, prescribing patterns, therapeutic indications, prophylactic recommendations, challenges of over-prescription and stewardship strategies that together shape responsible antibiotic use in dentistry.

## Current U.S. guidelines on antibiotic use in dentistry:

Antibiotics play an important role in treating dental infections, but unnecessary overprescribing by dental providers has resulted in serious antibiotic-related adverse effects. In response, several governing bodies in the U.S., including the American Dental Association (ADA), the American Academy of Orthopaedic Surgeons (AAOS) and the American Heart Association (AHA), have released clinical guidelines for dentists to follow. Despite these guidelines, the Centers for Disease Control (CDC) reports that about 10% of all outpatients' antibiotic prescriptions are written by dentists, with many not adhering to the recommended practices. The ADA, AHA, AAPD and CDC have consistently emphasized the value of prioritizing definitive dental treatment and limiting antibiotic prescriptions to cases where they are necessary. Following these recommendations would reduce the prevalence of adverse effects and guarantee patients' safety [8, 9]. In 2019, an expert panel convened by the American Dental Association (ADA) put forward an evidence-based report with recommendations for antibiotic prescribing when managing pulpal and periapical-related dental pain and intraoral swelling. This guideline parallels that published by the Academy of Pediatric Dentistry (AAPD) recommendations for antibiotic prescribing for children presenting with oral infections. Both U.S.-based institutions recommend avoiding the prescription of antibiotics when a definitive, conservative dental therapy is available. Instead, their use should be limited to situations where the dental infection shows signs of spreading or signs of a systemic infection. When prescribing, it is preferred that dentists provide a narrow-spectrum antibiotic. They stress the importance of antibiotic stewardship and highlight the adverse effects that could result from unnecessary prescriptions, such as *C. difficile* infection, allergic reactions and microbiome disruption [2, 10]. Patients with heart conditions and those who have received prosthetic joints have a higher chance of developing serious, life-threatening infections like infective endocarditis or prosthetic joint infection, respectively. Even though the risk was very low, many dentists would prefer to "play it safe" and choose to prescribe antibiotics. In 2007, the American Heart Association (AHA) published a guideline that it refined in 2021, endorsed by the ADA, restricting the use of antibiotics for high-risk cardiac patients. The guideline recommends that Amoxicillin should be used for procedures that manipulate gingival or periapical tissues or perforates the oral mucosa [11]. However, patients with prosthetic joints do not require antibiotic coverage according to a statement released by the ADA in collaboration with the American Academy of Orthopedic Surgeons (AAOS) as they failed to demonstrate an association between dental procedures and prosthetic joint infection. Individuals who are immunocompromised, have poorly controlled diabetes or have a history of joint infection are an exception and dentists are encouraged to confer with orthopedic specialists to direct clinical decision making [12].

## Common antibiotics prescribed in dental practice:

Dentists prescribed about 25.17 million systemic oral antibiotics, with β-lactam penicillin, specifically Amoxicillin, as the preferred choice according to data provided by the Journal of the American Dental Association (ADA) in 2022 [1]. This significant number places dentists fourth among prescribers of antibiotics, behind family practitioners, pediatricians and internists [13]. Amoxicillin, clindamycin, penicillin VK, azithromycin and amoxicillin-clavulanate were the top five antibiotics administered by American dentists, according to a Wisconsin-based study of dental antibiotic use from 2018 to 2021. These findings are consistent with normal clinical practice, which reserves clindamycin for patients with β-lactam allergies and favors narrow-spectrum penicillin when appropriate. Geographical disparities were also noted; dentists in the Northeast were more likely than those in the West to prescribe antibiotics. Additionally, female patients and older persons were shown to have higher prescribing rates [13]. Overall, antibiotic prescriptions by dentists have remained consistent over the years, with only minor changes in how those prescriptions are written. Mainly, between 2018 and 2022, a slight decrease in "days' supply" per prescription was observed, indicating a greater understanding of antibiotic stewardship principles. Another difference found is the decrease in the percentage of clindamycin prescriptions, even though it remains the main option for patients who report a penicillin allergy. This could be due to the increase in awareness of its connection to *Clostridium difficile* infection (CDI) [3]. Table 1 commonly prescribed Antibiotics in U.S Dental Practice. Dosing ranges are approximate based on typical dental prescribing patterns. Data summarized from studies and public health reports cited in text. These results demonstrate that amoxicillin is still the most often recommended antibiotic by dentists, with clindamycin and a few other frequently used substitutes following closely behind. It is easier to discuss the therapeutic and preventive uses of antibiotics as well as ways to improve stewardship in dental practice when these prescribing trends are acknowledged [14].

## Therapeutic use of antibiotics in common dental infections:

Current evidence-based landscape for the therapeutic use of antibiotics in common odontogenic infections emphasizes the critical role of dental practitioners in global Antimicrobial Stewardship (AMS). Being among the top prescribers of antimicrobials, dental professionals significantly influence the development of antimicrobial resistance (AMR) and must adhere strictly to current clinical guidelines [15].

## The definitive role of localized treatment:

According to current clinical guidelines established by organizations such as the American Dental Association (ADA) and the American Academy of Pediatric Dentistry (AAPD), systemic antibiotics are not indicated as the primary mode of therapy for most localized odontogenic infections in immunocompetent individuals [10]. Disease states-such as symptomatic irreversible pulpitis (SIP), pulp necrosis with symptomatic apical periodontitis (PN-SAP) and localized acute apical abscess (PN-LAAA)- are typically confined within the alveolar process. Therefore, the only definitive treatment involves Definitive Conservative Dental Treatment (DCDT), which includes surgical elimination of the microbial source through pulpal debridement, root canal therapy or incision and drainage (I & D) of an abscess. Systematic reviews confirm that for promptly treated localized infections, adjunctive antibiotic use provides no statistically significant improvement in clinical outcomes such as pain or swelling [16]. The unnecessary prescription of antibiotics in these situations contributes directly to AMR and represents a case of over-prescription [17].

## Indications for systemic therapy:

Systemic antibiotics are reserved for cases where the infection extends beyond local containment or poses a threat to patient health. Indications for antibiotic therapy include:

[1] Systemic involvement-Signs such as fever (≥100°F), malaise, trismus and lymphadenopathy

[2] Diffuse swelling (cellulitis)-The presence of indurated, non-fluctuant swelling in facial or intraoral regions, indicating infection spread to adjacent tissue planes.

[3] Compromised host immunity-Patients with systemic diseases (*e.g.*, uncontrolled diabetes or severe immunosuppression) whose resistance to infection is reduced.

When localized abscesses are present and DCDT must be deferred, a conditional or delayed prescription approach is preferred. The patient is instructed to initiate antibiotic therapy only if systemic symptoms worsen prior to receiving definitive treatment. This approach minimizes risk while upholding AMS principles [17].

## Empirical therapy regimens and duration:

Antibiotic therapy in dental infections is largely empirical, aimed at polymicrobial flora consisting of facultative and obligate anaerobes.

Treatment should utilize narrow-spectrum agents effective against common odontogenic pathogens.

[1] First-line agents: Amoxicillin (500 mg three times daily) or Penicillin VK (500 mg four times daily). In more severe cases, amoxicillin-clavulanate may be indicated.

[2] Penicillin allergy: Clindamycin (150-300 mg four times daily) is the preferred alternative, though it carries a risk of *Clostridioides difficile* infection.

The duration of antibiotic therapy should be limited to the shortest effective period-typically 3 to 7 days, depending on the initiation of DCDT and resolution of systemic signs [15]. Dental professionals are ethically and professionally obligated to follow evidence-based AMS practices. Adhering to guidelines that reserve antibiotics for cases involving systemic spread or compromised immunity protects both individual patients and public health. By prescribing antibiotics only as adjuncts to surgical source control, clinicians help preserve the efficacy of these essential medications [9]. Building upon the same principles of antimicrobial stewardship, it is equally important to apply evidence-based decision-making in situations where antibiotics are used for preventive rather than therapeutic purposes [18].

## Antibiotic prophylaxis in endocarditis and prosthetic joint patients:

Antibiotic prophylaxis helps prevent infective endocarditis (IE) and prosthetic joint infections that can occur after invasive dental procedures. Current recommendations limit its use to patients with conditions that place them at the highest risk of serious complications. Research over the past two decades shows that routine antibiotic use offers minimal added protection while contributing to resistance and adverse reactions. The shift toward targeted prevention and improved oral hygiene reflects a balance between patient safety and responsible antibiotic stewardship [19, 20]. Infective endocarditis (IE) is a potentially life-threatening infection of the heart's inner lining, most often caused by viridans group streptococci entering the bloodstream during transient bacteremia. Dental procedures that disturb gingival or periapical tissues can allow such microorganisms to enter circulation. Contemporary prevention strategies emphasize a risk-based approach to antibiotic use, underscoring the clinical rationale behind current recommendations [21].

## Current guidelines for IE prophylaxis:

According to the 2021 American Heart Association (AHA) scientific statement, antibiotic prophylaxis before dental procedures is reserved for clearly defined high-risk cardiac conditions. These include prosthetic heart valves or prosthetic material used in valve repair; a history of previous IE; specific congenital heart anomalies such as unrepaired cyanotic defects or repaired lesions with residual shunts or valvular regurgitation adjacent to prosthetic material; and cardiac transplant recipients with valvular disease. Prophylaxis is indicated only for dental procedures that manipulate gingival tissue, the periapical region or perforate the oral mucosa [11]. The standard drug regimen consists of amoxicillin 2 g orally, administered 30-60 minutes before the procedure. For patients allergic to penicillin, acceptable alternatives include cephalexin 2 g, azithromycin 500 mg or clarithromycin 500 mg. Clindamycin is no longer advised because of its higher association with adverse reactions and *Clostridioides difficile* infection [22].

## Prosthetic joint patients:

Joint guidelines from the American Dental Association (ADA) and American Academy of Orthopedic Surgeons (AAOS) do not recommend routine antibiotic prophylaxis for individuals with prosthetic joint implants undergoing dental procedures. Prophylaxis may be considered only after interdisciplinary consultation for patients who are immunocompromised, have poorly controlled diabetes or experienced postoperative complications [12]. This stance reflects the absence of strong evidence linking dental work to prosthetic joint infections and a growing recognition of antibiotic-related risks such as allergic reactions, gastrointestinal upset and the promotion of resistant bacterial strains [5].

## Rationale and risk-benefit considerations:

Poor oral hygiene has been shown to pose a greater risk of bacteremia and IE than dental treatment itself. Consequently, prevention now prioritizes daily oral care, patient-specific assessment, and close coordination between dental and medical professionals. Antibiotic prophylaxis should be reserved for patients truly at risk of IE or for select prosthetic joint recipients following professional consultation. Adhering to the AHA and ADA recommendations balances infection prevention with prudent antibiotic stewardship, aligning clinical practice with modern evidence-based standards. Since the 2007 revision of AHA guidelines, national IE incidence has remained stable or even declined- despite a major reduction in prophylactic antibiotic use [19, 21]. This trend suggests that widespread administration offers little protective advantage. Conversely, unnecessary exposure increases the likelihood of resistance, *C. difficile* infection [4] and hypersensitivity reactions [22].

## Challenges and consequences of antibiotic over-prescription in dentistry:

## Addressing antibiotic over-prescription:

Dentists often prescribe analgesics and antibiotics as part of post-operative management for controlling pain and treating odontogenic infections or related post-operative complications [16]. Though antibiotic prescriptions are routine in the field, several studies have found a significant proportion of those prescriptions to be unnecessary or inappropriate [23]. A dentist may follow common practice, educational training or literature and prescribe antibiotics as a precautionary measure. Still, the systemic over-issuance and over-use of antibiotics fuels a systemic risk in global antimicrobial resistance (AMR) and adverse drug events. The resulting financial burden on healthcare systems ultimately comes back to impact the patient [24].

## Contributing factors:

Overlapping or conflicting clinical symptoms are prevalent underlying challenges that surface as diagnostic uncertainty in dentistry. Distinguishing between bacterial infection classes is a primary example of the symptom-based challenges dentists face when prescribing for patients; some infection types require systemic therapy, while more typical inflammatory conditions may be managed with local treatment. Consequently, dentists often find themselves prescribing antibiotics "just in case" endodontic treatment is infeasible or when drainage may be an issue [25]. Commonly held public beliefs and patient expectations also help contribute to a widespread influence on post-procedural prescriptions. Patient and layman beliefs such as equating antibiotic prescription with quality post-procedure care are often a key driver in dentists choosing to prescribe them, sometimes at the cost of avoiding customer dissatisfaction, complaints and negative reviews [6]. Dental offices and dentists are frequently pressed for time and most dental offices lack advanced diagnostic tools. In urban areas, this leads to dentists not keeping up with continuing education and technological advances. Rural or underserved locations, on the other hand, frequently suffer from dentists adopting outdated local norms and educational resources. In either case, the lack of structured antimicrobial stewardship programs for dental practices on a national level perpetuates the feedback loop of habits that aren't audited or have clear data-driven sources [14].

## Unintended consequences:

The consequences of antibiotic over-prescription surface in clinical and public health settings as allergic responses, short-term gastrointestinal effects and sometimes life-threatening anaphylaxis. Studies have found nearly 4% of all patients receiving dental antibiotic prescriptions to have faced adverse effects, including hospitalization from *Clostridioides difficile* (*C. diff*) infections [8]. Dentists commonly prescribe Clindamycin and other broad-spectrum cephalosporins, often associated with *Clostridioides difficile* infections and gut microbiome disruptions. A "just in case" prescription has a nontrivial chance of endangering patient health and causing financial burden upon the patient and the healthcare system [24]. When observed at scale, dental antibiotic over-prescription and over-use accelerate the growing risk of global antimicrobial resistance, particularly among oral and systemic bacteria [26]. Routine dental procedures that depend on effective prophylaxis come under threat of losing efficacy in a growing trend of antimicrobial resistance, especially as resistant strains develop and spread across communities worldwide. Economically, the burden on both patients and healthcare systems compounds as unnecessary or inappropriate antibiotic prescriptions increase medication costs, follow-up care and adverse reaction treatments [27].

## Practical implications and solutions:

The answer is clear when looking at the problem from a systemic view - a lack of evidence-based antibiotic stewardship that incorporates regular and required clinical education, as well as clear auditing systems and mechanisms that hold dental practices nationwide accountable to a standard [27]. Several local treatment options, such as pulpectomies, drainage techniques and extractions, can often be utilized and promoted as definitive local treatments that avoid post-operative necessity for antibiotics, only achievable as a transformation at scale with structured stewardship programs [28]. Collaboration with public health officials and policy makers, pharmacists and infection control specialists and a structured public-private knowledge base for compliance will also drive a clearer adherence to set standards for prescription. Continuing education programs and stewardship/technological advancement integrations at dental offices are a key step in the required transformation. Digital prescription tracking systems collaborating with national stewardship digital frameworks can maintain accountability across the ecosystem and provide the needed data to observe and remedy over-prescription in geographical areas or even down to an individual dentist [29].

## Stewardship strategies and future directions in US dental practice:

Antibiotic stewardship has become an essential part of global efforts to control antimicrobial resistance (AMR). Dentists alone contribute around 10-12% of overall antibiotic prescriptions in the United States [26]. Although ADA has published updated guidelines, research shows that dentists have continued to over-prescribe, especially for pre-procedural prophylaxis, which is completely unnecessary. To address this issue, stewardship strategies are required to translate policies into actions. According to many studies, it is established that 80% of prophylactic antibiotics prescribed by dentists lack specific indications [30]. In many cases, prescription patterns highlight that decisions are influenced by patients' demand for antibiotics, diagnostic uncertainty, as well as habits rather than clinical evidence. A combination of stewardship strategies, such as education, monitoring and accountability, is critical for improving prescribing practices. The Centers for Disease Control and Prevention (CDC) introduced the 'Core Elements of Outpatient Antibiotic Stewardship' emphasizing commitment, policy implementation, tracking and education [7]. These ideas can be adapted for dental setups to aid prescriptions. For example, whenever prescriptions are provided without an appropriate diagnosis or an antibiotic duration exceeds recommendations, the electronic health record system can flag alerts [29]. Education is highly imperative for lasting enhancements in this matter. Continuing dental education should be interactive and case-based rather than a one-time lecture. Also, stewardship should be implemented in the undergraduate program curriculum early on [31]. However, surveys continue to show that dentists extend treatment durations and prescribe broad-spectrum agents unnecessarily [30]. A systematic approach through regulated feedback and peer comparison can help bridge this gap. The American Academy of Pediatric Dentistry (AAPD) continues to reinforce these stewardship principles. Its 2022 best practices document emphasizes that prophylaxis should be reserved for children at the highest risk of bacteremia induced complications and cautions that evidence in pediatrics is limited [18]. Robust evidence on how to change prescribing behavior is limited. A 2025 systematic review of dental antibiotic stewardship interventions identified three randomized controlled trials. Audit and personalized feedback combined with behavioral messaging consistently reduced unnecessary prescribing, whereas in person education alone had low certainty evidence and disseminating guideline summaries without interaction was ineffective [31]. Collaborate with other partners, such as the pharmacists, can help the dentists in choosing the most suitable antibiotic, its dosage and checking for allergies or interactions. Inter-professional association, supported by digital tools and national scrutiny, has already proven effective in medical settings and can bring parallel benefits to dentistry. In the future, a combination of technology, inter- professional teamwork and focus on related education can make antibiotic stewardship a routine part of any dental facility workflow [32].

## Conclusion:

A judicious approach is required in antibiotic prescription, which is solely based on evidence and prioritizes definitive local treatment while strictly adhering to guidelines from ADA, AHA, AAPD and CDC. Robust stewardship strategies through early and comprehensive education, monitoring and collaboration with pharmacists etc., are the need of the hour, when over-prescription continues to fuel antimicrobial resistance and adverse outcomes. Patient safety will only be ensured by integrating these principles into daily dental practices which will not only preserve antibiotic efficacy but also advance global public health.

## Figures and Tables

**Table 1:** Commonly prescribed Antibiotics in U.S dental practice

**Rank**	**Antibiotic Agent**	**Class**	**Typical dental dose**	**Notes**
1	Amoxicillin	Penicillin (β-lactam)	500 mg TID or 1g BID	First line for odontogenic infections in non-allergic patients.
2	Clindamycin	Lincosamide	300mg QID	Penicillin allergy alternative
3	Amoxicillin-clavulanate	Penicillin + β-lactamase inhibitor	875/125 mg BID	Broader coverage for mixed infection
4	Cephalexin	Cephalosporin (1st gen)	500 mg QID	Alternative when penicillin is contraindicated.
5	Penicillin VK	Penicillin	500mg QID	Narrow spectrum. Sometimes used for mild infections.
6	Azithromycin/Doxycycline/Metronidazole	Macrolide/Tetracycline/Nitroimidazole	Azithro: 500mg x 1 then 250mg daily. Doxy: 100mg BID Metro: 500mg TID	Used for Allergy, mixed anaerobic infection or periodontal therapy
*Dosing ranges
are approximate based
on typical dental
prescribing patterns.
